# Effect of Diffusion Limitations on Multianalyte Determination from Biased Biosensor Response

**DOI:** 10.3390/s140304634

**Published:** 2014-03-07

**Authors:** Romas Baronas, Juozas Kulys, Algirdas Lančinskas, Antanas Žilinskas

**Affiliations:** 1 Faculty of Mathematics and Informatics, Vilnius University, Naugarduko 24, Vilnius LT-03225, Lithuania; 2 Institute of Biochemistry, Vilnius University, Mokslininku 12, Vilnius LT-08662, Lithuania; E-Mail: juozas.kulys@bchi.vu.lt; 3 Institute of Mathematics and Informatics, Vilnius University, Akademijos 4, Vilnius LT-08663, Lithuania; E-Mails: algirdas.lancinskas@mii.vu.lt (A.L.); antanas.zilinskas@mii.vu.lt (A.Ž.)

**Keywords:** biosensor, quantitative analysis, mixture, modeling, simulation, noise, optimization

## Abstract

The optimization-based quantitative determination of multianalyte concentrations from biased biosensor responses is investigated under internal and external diffusion-limited conditions. A computational model of a biocatalytic amperometric biosensor utilizing a mono-enzyme-catalyzed (nonspecific) competitive conversion of two substrates was used to generate pseudo-experimental responses to mixtures of compounds. The influence of possible perturbations of the biosensor signal, due to a white noise- and temperature-induced trend, on the precision of the concentration determination has been investigated for different configurations of the biosensor operation. The optimization method was found to be suitable and accurate enough for the quantitative determination of the concentrations of the compounds from a given biosensor transient response. The computational experiments showed a complex dependence of the precision of the concentration estimation on the relative thickness of the outer diffusion layer, as well as on whether the biosensor operates under diffusion- or kinetics-limited conditions. When the biosensor response is affected by the induced exponential trend, the duration of the biosensor action can be optimized for increasing the accuracy of the quantitative analysis.

## 1. Introduction

Amperometric biosensors are analytical devices that measure the changes in the output current on the working electrode, due to the direct oxidation or reduction of the products of biochemical reactions [1,2]. The device specificity to particular analytes (substrates) is achieved by applying a biological material, usually an enzyme [3,4]. The calculation of analyte concentration from the biosensors signal (response) is rather simple in the case of a linear dependence of the signal on the substance concentration (linear calibration) [2,4]. The problem becomes more complex in the case of non-linear calibration or in the presence of mixtures of substances, producing the biosensors response [5–7].

Multianalytes have been successfully analyzed by biosensors using different multivariate approaches (e.g., partial least squares regression, principal component analysis) [8–10], artificial neural networks [11–15] and optimization approaches [16–18]. In those analytical systems, several enzymes or even several enzyme electrodes were used when components of the mixtures had an additive effect on biosensor response.

The multianalytes determination becomes even more complex if the biosensors response is perturbed by noise, e.g., white noise, sinusoidal power electrical noise or if the biosensor response is biased, e.g., by temperature change [19–21]. While in most electrochemical systems, the electrode noise and signal trend elimination are practically impossible, a comprehensive study of the background current provides valuable insight for analytical system design [19–21].

Recently, the problem of multianalytes determination by reverse problem solving has been explicitly formulated and successfully applied to optimize the calculation of multianalyte concentration using a response of a biocatalytic amperometric biosensor utilizing a mono-enzyme-catalyzed (nonspecific) conversion of multi-substrates [22]. The influence of the white noise-, as well as temperature-induced trend on the calculation of the analyte concentration has been also investigated at internal diffusion limitations by ignoring the external mass transport by diffusion. However, in practical biosensing systems, the diffusion of materials outside the enzyme region is of crucial importance for the biosensor response [23,24].

Real biosensors contain as an outer membrane a thin layer of porous or perforated polyvinyl alcohol, polyurethane, cellulose, latex or other material [2,4]. The outer membrane imposes an additional diffusion limitation to accessing the substrate to the catalytic layer and, therefore, increases the stability and prolongs the calibration curve of a biosensor. The external mass transport should be taken into consideration, even if no outer membrane is used and the Nernst diffusion layer approximation is applied [25,26]. In those cases, the mass transport outside the enzyme layer is usually modeled by an external diffusion layer [4,24–27]. In this work, the optimization-based method of the quantitative analysis of the biosensor response [22] is extended by taking into consideration the external mass transport.

The aim of this work is to investigate the effect of the external diffusion limitation on the precision of the determination of the multianalyte concentrations from the biased response of a biocatalytic amperometric biosensor utilizing a mono-enzyme-catalyzed multi-substrate conversion. The influence of the white noise-, as well as temperature-induced trend one the precision of multianalyte determination is also investigated. The investigation was carried out at very different catalytic conditions, thicknesses of the diffusion layer and types of signal noise.

Since a comprehensive analysis of a chemometric technique to be used for quantitative analysis usually requires a lot of input data, the biosensor responses to mixtures of compounds were simulated. On the other hand, computer simulation is usually much cheaper and faster than real experiments. Pseudo-experimental responses to mixtures of two compounds were numerically simulated using a computational model of the amperometric biosensor utilizing a mono-enzyme-catalyzed (nonspecific) conversion of two substrates [22]. The task of our investigation was to analyze the effect of temperature-induced biosensors response drift to analyte concentration calculation. Therefore, the simulated responses of the biosensor were perturbed by temperature-induced drift. The simulated responses to different concentrations of the substrates were used to extract the dependence of the transient output signal on the substrate concentrations. The resulting information was then used to determine the concentrations of the substrates from the testing simulated measurements. This work does not analyze the determination of individual analytes in the mixture. Rather, the concentrations of known different analytes were determined with the biosensor showing unspecific substrate conversion.

The numerical simulation is based on a two compartment mathematical model involving coupled reaction-diffusion equations [27,28]. The model comprises three regions: an enzyme layer, where enzymatic reaction, as well as the mass transport by diffusion takes place, a diffusion limiting region, where only the diffusion takes place, and a convective region. The numerical simulation was carried out using the finite difference technique [26,28,29]. The computational model of the biosensor was validated using known analytical solutions for mono-enzyme single substrate amperometric biosensors [27] and against published experimental data [30].

The numerical experiments showed that the precision of the concentration estimation significantly depends on the relative thickness (Biot number) of the outer diffusion layer. The accuracy of concentration estimation also depends on the biosensor response being under either the diffusion or the enzyme kinetics control, *i.e.*, on the diffusion module. The obtained dependencies can be applied in the development of smart analytical systems providing a high quality quantitative analysis of mixtures.

## 2. Mathematical Model

We consider a mono-enzyme dual biosensor (two-substrates) utilizing the Michaelis-Menten kinetics [1,3,30],

(1a) $$\text{E}+{\text{S}}_{1}\overset{k_{+1}}{\underset{k_{-1}}{⇌}}{\text{ES}}_{1}\overset{k_{3}}{\to }\text{E}+{\text{P}}_{1}$$

(1b) $$\text{E}+{\text{S}}_{2}\overset{k_{+2}}{\underset{k_{-2}}{⇌}}{\text{ES}}_{2}\overset{k_{4}}{\to }\text{E}+{\text{P}}_{2}$$

where E denotes the enzyme, S_1_ and S_2_ are the substrates to be determined, ES_1_, ES_2_ stand for the enzyme-substrate complexes, P_1_, P_2_ are the reaction products and *k*_+1_, *k*_−1_, *k*_+2_, *k*_−2_, *k*_3_, *k*_4_ are the kinetic constants.

When two substrates (S_1_ and S_2_) react with a single enzyme, E, without the formation of any two substrate complex, and the substrates do not combine directly with each other, then each substrate in a mixture of S_1_ and S_2_ acts as a competitive inhibitor of the others [31]. Particularly, 5*α*-androstan-3-one and 5*α*-androstane-3,16-dione are a special case of the substrates, S_1_ and S_2_, and 20*β*-hydroxy steroid-NADoxidoreductase (EC1.1.1.53) is a sample of the enzyme, E [30].

The reactions in the network given by Equation (1) are usually of different rates [1,31]. To sidestep the problems arising due to the large difference of timescales, the quasi-steady-state approach (QSSA) is often applied [30]. According to the QSSA, the concentration of the intermediate complex does not change with time; the reaction network (Equation (1)) reduces,

(2) $${\text{S}}_{1}+{\text{S}}_{2}\overset{\text{E}}{\to }{\text{P}}_{1}+{\text{P}}_{2}$$

**Figure 1.**
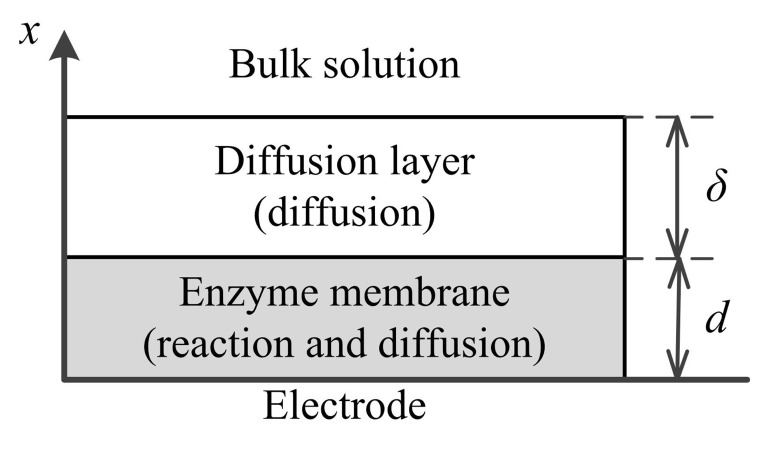
Principal structure of the biosensor.

The amperometric biosensor is treated as an electrode, and a relatively thin layer of an enzyme (enzyme membrane) is applied onto the electrode surface. The biosensor model involves three regions: the enzyme layer, where the biochemical reactions (Equation (1)), as well as the mass transport by diffusion takes place, the diffusion layer, where only the mass transport by diffusion of the substrates, as well as the products takes place, and a convective region, where the concentration of the substrates and products remains constant. Figure 1 shows the principal structure of the biosensor, where *x* = 0 represents the electrode surface and *x* = *d* corresponds to the boundary between the enzyme layer and the bulk solution; and, *δ* is the thickness of the external diffusion layer.

Assuming a symmetrical geometry of the electrode and a homogeneous distribution of the immobilized enzyme in the enzyme layer of a uniform thickness, the mathematical model of the biosensor action can be defined in a one-dimensional-in-space domain [26,27].

### 2.1. Governing Equations

Coupling the enzyme-catalyzed reactions given by Equation (1) in the enzyme layer with the one-dimensional-in-space diffusion leads to a system of the reaction-diffusion equations,

(3) $$\begin{array}{ll} \frac{\partial S_{i,e}}{\partial t} & =D_{S_{i},e}\frac{\partial^{2}S_{i,e}}{\partial x^{2}}-\frac{(V_{i}/K_{i})S_{i,e}}{1+S_{1,e}/K_{1}+S_{2,e}/K_{2}}\\ \frac{\partial P_{i,e}}{\partial t} & \begin{array}{cc} =D_{P_{i},e}\frac{\partial^{2}P_{i,e}}{\partial x^{2}}+\frac{(V_{i}/K_{i})S_{i,e}}{1+S_{1,e}/K_{1}+S_{2,e}/K_{2}} & \begin{array}{ccc} i=1,2 & 0<x<d, & t>0 \end{array} \end{array} \end{array}$$

where *x* and *t* stand for space and time, respectively, *S*_*i*,*e*_(*x, t*) and *P*_*i*,*e*_(*x, t*) are the molar concentrations of the substrate, S*_i_*, and the product, P*_i_*, in the enzyme layer, respectively, *V_i_* is the maximal enzymatic rate, *K_i_* is the Michaelis constant, *d* is the thickness of enzyme layer, *D_S_i,e__* and *D_P_i,e__* are the diffusion coefficients, *V*_1_ = *k*_3_*E*_0_, *V*_2_ = *k*_4_*E*_0_, *K*_1_ = (*k*_−1_ + *k*_3_)/*k*_+1_, *K*_2_ = (*k*_−2_ + *k*_4_)/*k*_+2_, and *E*_0_ is the total concentration of the enzyme, *i* = 1, 2.

Outside the enzyme layer, only the mass transport by diffusion of the substrates and products takes place,

(4) $$\begin{array}{lll} \frac{\partial S_{i,b}}{\partial t} & =D_{S_{i},b}\frac{\partial^{2}S_{i,b}}{\partial x^{2}}\\ \frac{\partial P_{i,b}}{\partial t} & =D_{P_{i},b}\frac{\partial^{2}P_{i,b}}{\partial x^{2}} & i=1,2\;d<x<d+\delta \;t>0 \end{array}$$

where *δ* is the thickness of the external diffusion layer, *S_i,b_*(*x*, *t*) and *P_i,b_*(*x*, *t*) are the concentrations of the substrate, S*_i_*, and the product, P*_i_*, in the bulk and *D_S_i,b__* and *D_P_i,b__* are the diffusion coefficients of the species in the bulk solution, *i* = 1,2.

### 2.2. Initial and Boundary Conditions

The biosensor operation starts when both substrates (S_1_ and S_2_) appear in the bulk solution (*t* = 0),

(5) $$\begin{array}{l} \begin{array}{ccc} S_{i,e}(x,0)=0, & P_{i,e}(x,0)=0, & 0\leq x\leq d \end{array}\\ \begin{array}{ccc} S_{i,b}(x,0)=0, & P_{i,b}(x,0)=0, & d\leq x<d+\delta \end{array}\\ \begin{array}{ccc} S_{i,b}(d+\delta ,0)=S_{i,0}, & P_{i,b}(d+\delta ,0)=0, & i=1,2 \end{array} \end{array}$$

where *S_i_*_,0_ is the concentration of the substrate, S*_i_*, in the bulk solution, *i* = 1,2.

At the electrode surface (*x* = 0), due to the electrode polarization, the concentrations of the reaction products (P_1_ and P_2_) are permanently reduced to zero, while for the non-ionized substrates, their fluxes are assumed to be zero (*t* > 0) [27],

(6) $$\begin{array}{cc} {\begin{array}{cc} P_{i,e}(0,t)=0, & D_{S_{i},e}\frac{\partial S_{i,e}}{\partial x}| \end{array}}_{x=0}=0, & i=1,2 \end{array}$$

Assuming a well-stirred buffer solution leads to the constant thickness of the diffusion layer, as well as the constant concentration above that layer during the biosensor operation (*t* > 0),

(7) $$\begin{array}{ccc} S_{i,b}(d+\delta ,t)=S_{i,0}, & P_{i,b}(d+\delta ,t)=0, & i=1,2 \end{array}$$

On the boundary between two adjacent layers, the merge conditions are defined for the substrates, as well as the products (*t* > 0),

(8) $$\begin{array}{ll} {D_{S_{i},e}\frac{\partial S_{i,e}}{\partial x}|}_{x=d}=D_{S_{i},b}{\frac{\partial S_{i,b}}{\partial x}|}_{x=d}, & S_{i,e}(d,t)=S_{i,b}(d,t)\\ {D_{P_{i},e}\frac{\partial P_{i,e}}{\partial x}|}_{x=d}=D_{P_{i},b}{\frac{\partial P_{i,b}}{\partial x}|}_{x=d}, & P_{i,e}(d,t)=P_{i,b}(d,t) \end{array}$$

The diffusion layer (*d* < *x* < *d* + *δ*) may be treated as the Nernst diffusion layer [28]. According to the Nernst approach, the diffusion layer of the thickness, *δ*, remains unchanged with time.

### 2.3. Biosensor Response

The measured anodic or cathodic current is usually assumed as the response of an amperometric biosensor. When modeling amperometric biosensors, due to the direct proportionality of the current to the area of the electrode surface, the current is often normalized with that area [26,27]. The density, *I*(*t*), of the biosensor current at time *t* depends upon the fluxes of the both products at the electrode surface and can be expressed explicitly from the Faraday and the Fick laws [31],

(9) $$\begin{array}{cc} I(t)=n_{1}FD_{P_{1,e}}{\frac{\partial P_{1,e}}{\partial x}|}_{x=0}+n_{2}FD_{P_{2,e}}{\frac{\partial P_{2,e}}{\partial x}|}_{x=0}, & I_{\infty }=\underset{t\to \infty }{\lim} \end{array}I(t)$$

where *n*_1_ and *n*_2_ are the numbers of electrons involved in charge transfers in the corresponding electrochemical reactions at the electrode surface, *I*_∞_ is the density of the steady-state biosensor current and *F* is the Faraday constant, *F* = 96, 486 C/mol.

### 2.4. Characteristics of Biosensor Action

The diffusion module or Damköhler number essentially compares the rate of the enzyme reaction (*V_i_*/*K_i_*) with the mass transport through the enzyme-loaded layer (*D_S_i,e__*/*d*^2^) [27,32],

(10) $$\begin{array}{cc} \Phi_{i}^{2}=\frac{V_{i}d^{2}}{K_{i}D_{S_{i,e}}}, & i=1,2 \end{array}$$

where $\Phi_{i}^{2}$ is the dimensionless diffusion module corresponding to the *i*—th reaction of the network given by Equation (1).

If the diffusion module is less then unity, then enzyme kinetics (reaction rate) controls the biosensor response. The response is controlled or limited by diffusion when the module is greater than unity [27]. At intermediate values of the diffusion module, the biosensor operation is of a mixed control. The quantitative analysis of mixtures may notably depend on whether the biosensor response is under diffusion or enzyme kinetics control [12,13,22].

The Biot number is another dimensionless parameter widely used to indicate the internal mass transfer resistance to the external one [4,33],

(11) $$\begin{array}{cc} \beta_{i}=\frac{d/D_{S_{i},e}}{\delta /D_{S_{i},b}}=\frac{dD_{S_{i},b}}{\delta D_{S_{i,e}}}, & i=1,2 \end{array}$$

where *β*_1_ and *β*_2_ are two Biot numbers corresponding to the diffusion of two substrates.

### 2.5. Signal Trend and Noise

In real-life experiments, the reaction rate can be affected by the temperature change that induces a trend of the output signal of the sensor [19,34]. The trend factor, *R*(*t*), is often expressed by the Arrhenius equation, which defines the dependency of the reaction rate on the temperature [35]. The trended signal, *I_T_*(*t*), can be expressed as the multiplication of the initial output, *I*(*t*), by a trend factor, *R*(*t*), *t* > 0,

(12) $$I_{T}(t)=I(t)\times R(t)$$

Assuming that the temperature, *T*, changes linearly with time (*T* = *T*_0_ + *at*) and *R*(0) equals unity, the trend factor, *R*(*t*), is expressed as follows:

(13) $$R(t)=\exp\left(\frac{E_{a}}{\bar{R}}\times \frac{at}{T_{0}(T_{0}+at)}\right)$$

where *E_a_* is the activation energy, *R̄* = 1.98 (cal/(K · mol)) is the gas constant, *T* is absolute temperature, *T*_0_ = 298 K, and *a* (K/s) is the coefficient of proportionality [22,35]. The zero value of *a* corresponds to a signal effected with no trend, *I_T_* (*t*) = *I*(*t*) at *a* = 0 and *t* > 0.

Besides the signal trend, the biosensor response can be also affected by an unpredictable noise [20,21]. The measurements, *I_N_* (*t*), of the noisy signal can be modeled by adding a white Gaussian noise to the noise free biosensor response, *I* (*t*),

(14) $$I_{N}(t)=I(t)+\xi$$

where ξ ∼ 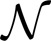
(0,*σI*(*t*)) stands for the random number, generated following the Gaussian distribution with zero mean and standard deviation equal to *σI*(*t*) [22]. Then, the trended noisy output signal *I_TN_*(*t*) can be modeled as follows:

(15) $$I_{TN}(t)=I(t)R(t)+\xi$$

The noise can be signal-dependent or signal-independent [20]. The electrical background noise is one of the major limitations of the amperometric detection of the smallest possible concentrations of electrochemically active materials [36]. The background noise is usually signal independent. This work concentrates on the determination of relatively high concentrations of substrates in comparison with the corresponding Michaelis constant. The multiplicative signal-dependent noise is used in the modeling, since the measurement errors in such applications are usually proportional to the measured signal value [20,36]. The influence of the additive noise can be also investigated; e.g., the influence of both kinds of noise was compared in [22] for a similar model, but without an external diffusion layer.

### 2.6. Numerical Simulation

Due to the nonlinearity of the governing Equation (3), the initial boundary value problem, Equations (3)–(8), can be analytically solved only for a very specific set of the model parameters [27,28]. Because of this, the problem, Equations (3)–(8), was solved numerically by applying the finite difference technique [26,28,29]. In the space coordinate, *x*, the enzyme and the external diffusion layers were divided into the same number of subintervals of equal length. A uniform discrete grid was also introduced in the time coordinate, *t*. An explicit finite difference scheme has been built as a result of the difference approximation of Equations (3)–(8) [22,29]. The numerical simulator of the biosensor action has been programmed in the C++ language [37].

Explicit difference schemes have a convenient algorithm of the calculation and are simple for programming [26,28]. The explicit scheme used for Equations (3)–(8) was conditionally stable and converges with the rate *O*(*τ* + max(*h*_1_,*h*_2_)^2^), where *h*_1_ = *d*/*N, h*_2_ = *δ*/*N*. In the simulation, both layers were discretized with the same number *N* = 200 of grid points. *N* was constant in the simulation of all the responses, while the time step size, *τ*, was recalculated for each simulation. To make the difference scheme stable, the step size, *τ*, was found from the sufficient stability conditions, $\tau \max(D_{S_{i},e},D_{P_{i},e})\leq h_{1}^{2}/4$, $\tau \max(D_{S_{i},e},D_{P_{i},e})\leq h_{2}^{2}/2$, *i* = 1, 2, and *τ* max (*V*_1_, *V*_2_) ≤ (*K*_1_ + *K*_2_)/2 [26,38].

The mathematical, as well as the corresponding computational models of the biosensor were validated using known analytical solutions for mono-enzyme single substrate amperometric biosensors [27]. When the concentration of the substrates is low in comparison with the corresponding Michaelis constant (*S_i,e_* < *S_i_*_,0_ ≪ *K_i_*), the nonlinear reaction terms in Equation (3) simplify to those of the first order, (*V_i_*/*K_i_*)*S_i_*, *i* = 1, 2.

For validating the model in the opposite case of the substrate concentrations, a high concentration, *S_i_*_,0_, of the substrate, S*_i_*, was used together with a zero concentration of another substrate, *S*_1,0_ ≫ *K*_1_, *S*_2,0_ = 0 and *S*_2,0_ ≫ *K*_2_, *S*_1,0_ = 0. In both these cases of the extreme concentrations of the substrates, the initial boundary value problem, Equations (3)–(8), reduces to a linear one and can be solved analytically [22,26,27]. Reducing the thickness of the diffusion layer (*δ* → 0), the two compartment model, Equations (3)–(8), approaches a known one compartment model [22], the numerical solution of which was also used for validating the numerical simulation of the problem, Equations (3)–(8).

The calculated values of the reaction term Equation (3), as of the total rate of Reaction (1) at steady-state conditions was also validated using published experimental data [30], accepting 5*α*-androstan-3-one and 5*α*-androstane-3,16-dione as the substrates, S_1_ and S_2_, and 20*β*-hydroxy steroid-NAD oxidoreductase (EC 1.1.1.53) as the enzyme, E.

The following values of the model parameters were constant in all the numerical experiments:

(16) $$\begin{array}{l} \begin{array}{cc} D_{S_{i},e}=D_{P_{i},e}=3\times 10^{-6}{\mathrm{cm}}^{2}/\text{s}, & D_{S_{i},b}=D_{P_{i},b}=6\times 10^{-6}{\mathrm{cm}}^{2}/\text{s} \end{array}\\ \begin{array}{cccc} K_{i}=10^{-4}\text{M}, & d=0.02\mathrm{cm}, & n_{i}=1, & i=1,2 \end{array} \end{array}$$

The membranes were characterized by a diffusion coefficient of substrates. For the calculation, the diffusion coefficient, 3 × 10^−6^ cm^2^/s, was used, which is typical for the enzyme membranes of biosensors and is two times less than that in the buffer solution [39].

In order to investigate the impact of the external diffusion limitation on the precision of the determination of the multianalyte concentrations and to avoid results dependent on the predefined values Equation (16), the biosensor response was simulated at different values of the thickness, *δ*, of the diffusion layer chosen, so that the Biot numbers Equation (11) would vary in a wide range (from 0.4 up to ∞). The infinite Biot number corresponds to the zero thickness of the diffusion layer or to the extremely low diffusivity of materials in the diffusion layer. Although the thickness, *δ*, of the diffusion layer was varied in the investigation, similar responses could be also simulated by changing the diffusion coefficients, *D_S_i_,b_* and *D_P_i_,b_* (*i* = 1, 2), of materials in the external diffusion layer, keeping the same Biot numbers. To minimize the results dependent on the catalytic properties of the biosensor, the response was simulated at different value of diffusion modules Equation (10) by changing the maximal enzymatic rates, *V*_1_ and *V*_2_, so that the response would be in both enzyme kinetics and diffusion limitations.

The numerical solutions of the model, Equations (3)–(8), were compared with the analytical solutions at different values of *V_i_* and *S_i_*_,0_, *i* = 1,2. The relative difference between the numerical and analytical solutions was less than 1% when applying the stability conditions defined above.

The rate of the trend is characterized by the activation energy, *E_a_*, and the coefficient, *a*, of proportionality The simulated biosensor responses were affected by different trends using different values of the latter parameters. Values of the trend parameters used in the calculations are presented in Table 1, where the first column stands for the denotation of the trend, the second one for the value of activation energy, *E_a_* and the last column for the value of the coefficient, *a*, of proportionality These values were selected to be the same as in [22] and to model trends similar to the temperature-induced experimental ones [40].

**Figure 2.**
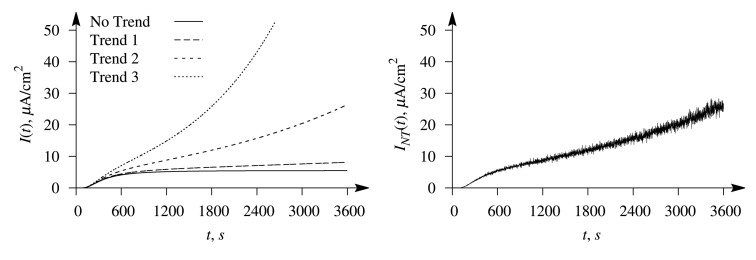
The dynamics of the biosensor current affected by the exponential trends (**Left**) and in conjugation with the white Gaussian noise (**Right**, *σ* = 0.05) simulated at the following values of the model parameters: *V*_1_ = 0.5 μM/s, *V*_2_ = 5 μM/s, *S*_1,0_ = 0.32 mM, *S*_2,0_ = 1.28 mM. The other parameters are as defined in Equation (16).

The evolution of the typical biosensor responses is illustrated in Figure 2, where the left plot shows the signals affected by the exponential trend of a different rate and the right plot the response affected by the trend, entitled Trend 2, in conjugation with the white Gaussian noise at *σ* = 0.05. The simulation was performed at the maximal enzymatic rate *V*_1_ = 0.5 μM/s for the first substrate and *V*_2_ = 10 V_1_ = 5 μM/s for the second one; the concentration of the first substrate was *S*_1,0_ = 3.2*K*_1_ = 0.32 mM and *S*_2,0_ = 4 *S*_1,0_= 12.8*K*_2_ = 1.28 mM of the second substrate, and the thickness of the outer diffusion layer was *δ* = 1 mm. Values of the other parameters were as defined in Equation (16). At the values used in the simulation, the Biot numbers were *β*_1_ = *β*_2_ = 0.4 and the diffusion modules: $\Phi_{1}^{2}\approx 0.67$, $\Phi_{2}^{2}=10\Phi_{1}^{2}\approx 6.7$.

## 3. Optimization Problem

We consider the quantification of mixtures of two substrates from the response of the mono-enzyme biosensor. Similar problems for simpler models have been already considered in [18,22]. In this work, we consider the quantitative determination of two substrates acting as competitive inhibitors of each other at the external diffusion limitation. As in [22], the measurements are supposed to be corrupted by a white Gaussian noise, and the reaction rate is to be affected by the temperature variance [7,19]. Pseudo-experimental simulated data were used for investigating the effect of diffusion limitations on the the precision of the concentration estimation. A similar approach to using simulated biosensor responses has been applied for training a neural network to perform quantitative analysis of the mixtures [12,13].

### 3.1. Statement of the Optimization Problem

Let *W* = (*w*_1_,…, *w_n_*) be a sequence of test measurements of the biosensor current at discrete time moments, *t*_1_, …, *t_n_*, and let *Z*(*c*) = (*z*(*t*_1_, *c*), …, *z*(*t_n_*, *c*)) be a sequence of the corresponding values of the response numerically simulated using the model, Equations (3)–(8), where *c* = (*c*_1_, *c*_2_) stands for the concentrations of the substrates, S_1_ and S_2_, which are the subject to be evaluated, and *z*(*t_i_*, *c*) is the simulated biosensor current at time moment *t_i_*, *i* = 1, …, *n*.

The concentrations c = (*c*_1_, *c*_2_) can be evaluated by tuning the values of the concentrations with respect to the conformity of theoretical measurements, *Z*(*c*), to the given sequence, *W*, of the measured biosensor currents. In order to perform the tuning, the least squares approach has been applied in [18], where the relevant problem has been described as:

(17) $$\tilde{c}=\arg\underset{c\in C}{\min}\overset{n}{\underset{i=1}{\text{∑}}}{\left(z(t_{i},c)-w_{i}\right)}^{2}$$

where *C* denotes the feasible region of the values of concentrations *c* = (*c*_1_, *c*_2_).

When the biosensor output is affected by the exponential trend Equation (13), the mathematical optimization problem given by Equation (17) should be reformulated as:

(18) $$(\tilde{c},\tilde{a})=\arg\underset{\left\{c\in C,a_{-}\leq a\leq a_{+}\right\}}{\min}\overset{n}{\underset{i=1}{\text{∑}}}{\left(z(t_{i},c)R(t_{i})-w_{i}\right)}^{2},$$

where the decision parameter, *a*, is supposed to be unknown in Equation (13) and the subject is to be evaluated within the feasible interval [*a*_−_, *a*_+_].

The optimization problem defined in Equation (18) is a generalization of the optimization problem given by Equation (17), and both of them coincide when *R*(*t*) = 1 or; in other words, when *E_a_* = 0 and/or *a* = 0 in Equation (13).

### 3.2. Solution of the Optimization Problem

The optimization problems stated above are difficult to solve, due to mathematically unprovable convexity and uni-modality properties of the objective function, thus limiting the applicability of local optimization algorithms, and are highly computational resource-consuming to find a single value of the objective function. The approach to apply a surrogate objective function has been proposed in [22], where the surrogate objective function, *z̃*(*t_i_*, *c*), is defined as bi-linear interpolant of the function, *z*(*t_i_*, *c*), assuming that the theoretical biosensor responses, *z*(*t_i_*, *c*), are already simulated using a discrete set, *C′*, of concentrations *c* = (*c*_1_, *c*_2_) for all *i* = 1, …, *n*.

In the optimization problem given by Equation (18), the objective is the squared error of the measured biosensor response (*w*_1_, …, *w_n_*) at discrete time moments *t*_1_, …, *t_n_* with respect to known (simulated or measured experimentally) biosensor responses *Z*(*c*) obtained at the feasible region, *C*, of the substrate concentrations. The decision variables are the concentrations, *c*_1_ and *c*_2_, to be determined, and the feasible region, *C*, is defined by their intervals. For the optimization, a state-of-the-art algorithm was used [22]. The maximal time, *t_n_*, is chosen so large that all the biosensor responses, *Z*(*c*), approach the steady state theretofore.

The feasible region for the concentrations *c* = (*c*_1_, *c*_2_) has been chosen to be *C* = [3.2, 12.8]^2^, assuming that the concentrations are dimensionless and normalized with respect to the corresponding Michaelis constant as follows: *c_i_* = *S_i_*_,0_/*K_i_*, *i* = 1, 2. The biosensors calibration curve is usually linear at the substrate concentrations lower than the Michaelis constant, and then, the determination of the analyte concentration from the biosensor response is rather simple [1,2,4]. Because of this, the feasible region was chosen so that *S_i_*_,0_ > *K_i_*, *i* = 1, 2, when the concentration determination is more complex.

The entire domain, *C*, has been discretized with the uniform grid with a step size equal to 0.1, the nodes of which (97^2^ = 9, 409 in total) comprise the discrete set, *C′*. The theoretical biosensor response values, *z*(*t_i_*, *c*), have been computed for every combination, (*c*_1_, *c*_2_) ∈ *C′*, assuming that *t*_0_ = 0; *t_i_*_+1_ − *t_i_* = 1 s; *i* = 1,…, *n* − 1; *n* = 3, 600. Such a predefined duration (*t_n_* = 3, 600 s) of the biosensor operation is sufficient to reach the steady-state current using the thickest diffusion layer and lowest reaction rate that was used in the computational experiments.

The computation of a pseudo-experimental response *Z*(*c*) = (*z*(*t*_1_, *c*),…, *z*(*t_n_*, *c*)) for a particular pair *c* = (*c*_1_, *c*_2_) of the concentrations requires from around 15 to around 35 min (depending on the thickness of the diffusion layer and the reaction rate) using one core of an Intel Xeon X7550 (2 GHz) processor. Therefore, the computation of the responses for the whole set, *C′*, of the concentrations is time consuming or even impossible within reasonable time. On the other hand, the simulation of different biosensor responses can be considered as independent tasks and performed in parallel by assigning them to different computing resources. For this purpose, the framework for computational simulation of a large set of biosensor responses using high performance computing facilities has been developed. The framework is based on the master-slave strategy, where one of the processing units (the master) is considered to be responsible for the management of the queue of simulations assigned to be performed, and the others (the slaves) performs computational work assigned by the master [41]. The framework has been used on a high performance facility, consisting of 32 Intel Xeon X7550 processors with eight cores each (256 cores in total). Due to the homogeneity of the processing units and the relatively small costs required for communication between them, the speedup of the computations was almost linear (almost equal to the number of processing units that were used), thus allowing one to simulate the responses, *Z*(*c*), for a whole set, *C′*, of pairs *c* = (*c*_1_, *c*_2_) of the concentrations within reasonable time.

Since the simulation of a single biosensor response requires from 15 up to 35 min when using a single core of an Intel Xeon X7550 processor, the simulation of 9, 409 such responses would require more than 2, 352 h, whereas the latter computations using 256 cores of Intel Xeon X7550 processor require less than 10 h. This indicates the almost linear speed-up of the computations or, in the other words, a reduction of the runtime by almost 256 times.

The obtained pseudo-experimental responses, *Z*(*c*) (*c* ∈ *C′*), have been used to derive the values of the surrogate objective function, *z̃*(*t*, *c*), by applying the bi-linear interpolation approach. Because of the availability of the analytical expression of the gradient of *z̃*(*t*, *c*), a standard gradient descend method can be applied to minimize *z̃*(*t*, *c*).

The optimization-based method for the evaluation of the concentrations has been implemented in a MATLAB environment using the Multi-Startstrategy with an efficient local minimization function, *fmincon*, which is included into the optimization toolbox as a subroutine [42]. A user-defined formula was used for the gradients of the objective functions, Equations (17) and (18). The local search has been performed three times using different initial solutions, randomly generated within the feasible region.

The simulation of the biosensor responses is much more time-consuming than the optimization process; therefore the response simulator has been programmed in C++, including distributed memory parallel programming libraries.

The standard stopping criteria for the local search has been defined by a tolerance *Tol Fun* = 10^−5^ of the function value [42]. Since the scale of the biosensor response varies depending on the model parameters, the same function value corresponds to the different discrepancy between the simulated and the given measurements of the biosensor response. To unify the scale of the biosensor response and the scale of the objective function values, the measurements of the biosensor response have been multiplied by a scalar, chosen with respect to making the maximal objective function value equal to 10^5^.

## 4. Results and Discussion

The optimization-based method for the evaluation of the concentrations (see Section 3.2) has been used to investigate the precision of the evaluation of the concentrations, when the biosensor response follows the model, Equations (3)–(9). Each component of the mixture was characterized by the individual maximal enzymatic rate differing in an order of magnitude. Without reducing the generality, it was assumed that the maximal enzymatic rate, *V*_2_, for the second substrate is greater than the rate, *V*_1_, for the first one, *V*_2_ = 10*V*_1_ and $\Phi_{2}^{2}=10\Phi_{1}^{2}$. Similar approaches have been already used in a quantitative analysis of mixtures in [12,13,22].

The quality of the quantitative analysis is influenced by whether the biosensor response is under the diffusion or the enzyme kinetics control, and the concentration estimation is usually more accurate for a substrate corresponding to a greater diffusion module than for another substrate corresponding to a lower diffusion module [12,13,22]. Because of this, the maximal enzymatic rates, *V*_1_ and *V*_2_, were chosen, so that the biosensor response can be controlled by the enzyme kinetics for the first component ( $\Phi_{1}^{2}<1$) and by the mass transport for the second one ( $\Phi_{2}^{2}>1$), *V*_1_ = 0.5 μM/s, *V*_2_ = 10 *V*_1_ = 5 μM/s, $\Phi_{1}^{2}\approx 0.67$, $\Phi_{2}^{2}=10\Phi_{1}^{2}\approx 6.7$.

**Figure 3.**
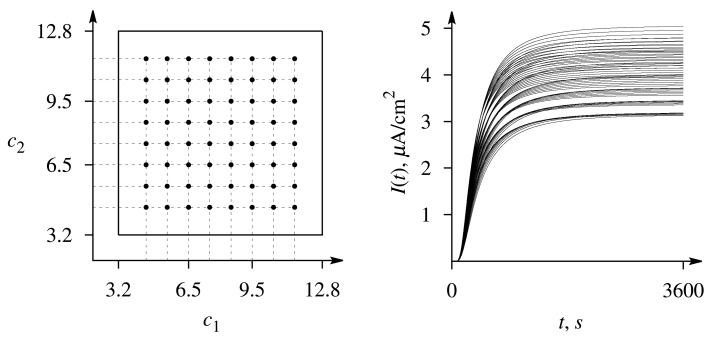
The set of the dimensionless concentrations, *c*_1_ and *c*_2_, of the substrates, S_1_ and S_2_, used in the investigation, as well as the noise-free biosensor responses to these concentrations simulated at and *V*_1_ = 0.5 μM/s, *V*_2_ = 5 μM/s and *β* = 0.4.

The precision of the evaluation has been determined by attempting to evaluate the set of 64 pairs *c* = (*c*_1_, *c*_2_) of predefined concentrations for which the responses were simulated (see Figure 3) and calculating the average precision of the estimates *c̃* = (*c̃*_1_, *c̃*_2_) obtained by the optimization-based method. The precision of a single estimate of the concentration has been determined by the relative error,

(19) $$\varepsilon_{i}=\frac{\left|c_{i}-{\tilde{c}}_{i}\right|}{c_{i}},i=1,2$$

**Figure 4.**
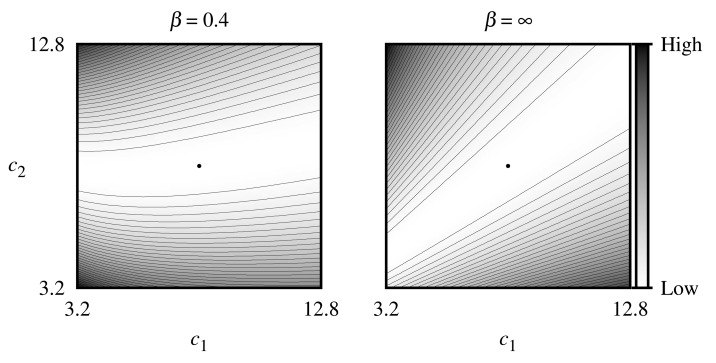
Contour lines of the objective function given by Equation (17) for the noise-free biosensor responses simulated at *V*_1_ = 0.5 μM/s, *V*_2_ = 5 μM/s assuming zero (*β* = ∞) and a relatively thick (*β* = 0.4) thickness of the external diffusion layer, (*c*_1_, *c*_2_) = (8, 8).

The sensitivity of the estimates of the concentrations is illustrated by Figure 4, where contour lines of the objective function given by Equation (17) for the noise-free biosensor responses were simulated at *V*_1_ = 0.5 μM/s, *V*_2_ = 5 μM/s assuming zero thickness (*β* = ∞) of the external diffusion layer, as well as the relatively thick (*β* = 0.4) diffusion layer, where the true dimensionless concentrations (*c*_1_, *c*_2_) of the substrates were equal to (8, 8). As one can see in Figure 4, the objective function for both values of *β*-parameter is unimodal in the region of interest, and the minimum point lies in a wide flat valley, which is curved and can negatively influence the convergence of the gradient-based algorithm when *β* = 0.4.

The maximal time *t_n_* = 3, 200 s until which the responses were measured was chosen so large that all the biosensor responses approach the steady state. Probably, a reasonable quality of the concentration determination can be achieved from shorter responses when the steady state is not reached. In order to investigate the influence of the duration of the biosensor operation on the precision of the concentration evaluation, the concentration evaluation has been performed by analyzing biosensor responses of different duration *t_m_* of the biosensor operations;, *t_m_* ∈ {200, 400, 800, 1, 600, 3, 200}, first seconds of the operation, *t_m_* ≤ *t_n_*. The duration *t_m_* = *t_n_* = 3,200 s corresponds to the steady-state response.

In order to investigate the impact of the thickness, *δ*, of the external diffusion layer, the biosensor responses were simulated at different values of *δ* chosen so that the Biot number *β* (*β* = *β*_1_ = *β*_2_) would vary in a wide range (from 0.4 up to ∞), assuming the thickness, *d*, of the enzyme layer to be constant, as defined in Equation (16). The infinite Biot number (*β* = ∞) corresponds to the zero thickness of the diffusion layer.

Since the concentrations from the noise-free biosensor responses can be evaluated very precisely [13,18] and the measurements in real experiments are often affected by an unpredictable noise [19–21], only the noisy responses modeled by adding either an entirely white Gaussian noise Equation (14) or that noise in conjugation with the induced trend Equation (15) were used for investigating the precision of the concentration evaluation.

### 4.1. Impact of Biosensor Response Time

The response time is an important characteristic of biosensors. In various applications, it is important to have the response time as short as possible [1,3]. The response time of enzymatic biosensors depends to a great extent on the diffusion processes [4,26]. Particularly, an increase in the thickness of the external diffusion layer prolongs the steady-state response time. When the steady-state response time is relative large the biosensor current measured much earlier than the steady-state achieved can be used as the biosensor response. Having only partial data on the evolution of the biosensor current can notably decrease the precision of the quantitative analysis of the analyte [22]. Because of this, it is important to know how short the duration of the biosensor operation can be for the substrate concentrations to be suitably evaluated.

**Figure 5.**
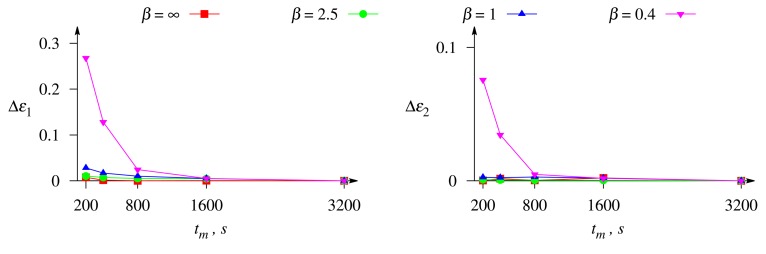
The impairment, Δ*ε_i_*, of the relative error, *ε_i_*, of the evaluation of the concentration, *c_i_*, of the substrate, S*_i_*, *versus* the duration, *t_m_*, of the biosensor operation at four values of the Biot number, *β*, when the biosensor response is affected by white Gaussian noise with *σ* = 0.05, *i* = 1,2. The other parameters are as in Figure 2.

Computational results showed that for all practical values of the thickness, *δ*, of the outer diffusion layer, the concentrations can be evaluated with a relative error lower than 0.05 from the measurements of the longest biosensor response (*t_m_* = 3, 200 s) corrupted by the white Gaussian noise with *σ* = 0.05 (see Equation (14)). However, as one can see in Figure 5, the precision can be impaired using measurements of a shorter biosensor response. In Figure 5, different curves stand for the different thicknesses of the diffusion layer, corresponding to the different Biot numbers. The impairment of the precision, when shortening from *t_n_* to *t_m_* of the biosensor response, has been evaluated by the change of the relative error:

(20) $$\Delta \varepsilon_{i}(t_{m})=\varepsilon_{i}^{\left(m\right)}-\varepsilon_{i}$$

where *ε_i_* is the relative error of the estimate of the concentration, *c_i_*, obtained using measurement of the first *t_n_* seconds of the biosensor response (see Equation (19)), and $\varepsilon_{i}^{\left(m\right)}$ is the relative error obtained using measurements of the first *t_m_* seconds of the biosensor response, *i* = 1, 2. The reference value *t_n_* = 3, 200 corresponds to the steady-state response.

Figure 5 shows that shortening of the biosensor response time, *t_m_*, from 3,200 down to 800 s, the relative error (Δ*ε_i_*, as well as *ε_i_*) of the evaluation of the substrate concentrations arises insignificantly or even remains practically the same (e.g., when *β* = ∞). At relatively short-term biosensor responses (*t_m_* = 200 and *t_m_* = 400 s), the impairment, Δ*ε_i_*, of the relative error notably increases with decreasing the Biot number, *β* (increasing the thickness *δ* of the diffusion layer). The impact of the Biot number on the accuracy of the concentration evaluation is discussed in detail below.

Since the maximal enzymatic rate of the second substrate was notably (ten-fold) greater than of the first one, the second substrate has more impact on the biosensor response [22,26]. Moreover, the concentration, *c*_2_, of the second substrate was evaluated notably more precisely than that of the first one in all the numerical experiments that were performed. Taking these considerations into account, bellow, the investigation is focused on the evaluation only of the concentration, *c*_1_, as less precisely determined.

### 4.2. Impact of Exponential Signal Trend

Similar computational experiments were applied for investigating the impact of thickness *δ* of the outer diffusion layer in conjugation with the temperature-induced trend. The results showed that the concentrations of both competitive substrates can be precisely evaluated practically independent of the thickness of the diffusion layer, the duration of the biosensor operation and the rate of the exponential trend, when the noise-free biosensor responses are analyzed.

The maximal relative errors, *ε*_1_ and *ε*_2_, of the concentrations, *c*_1_ and *c*_2_, were less than 0.02 and 0.005, respectively. The most intractable situations occurred when the diffusion layer was the thickest (*β* = 0.4), the biosensor response was shortest (*t_m_* = 200 s) and the signal was affected by the exponential trend of the highest rate (Trend 3).

**Figure 6.**
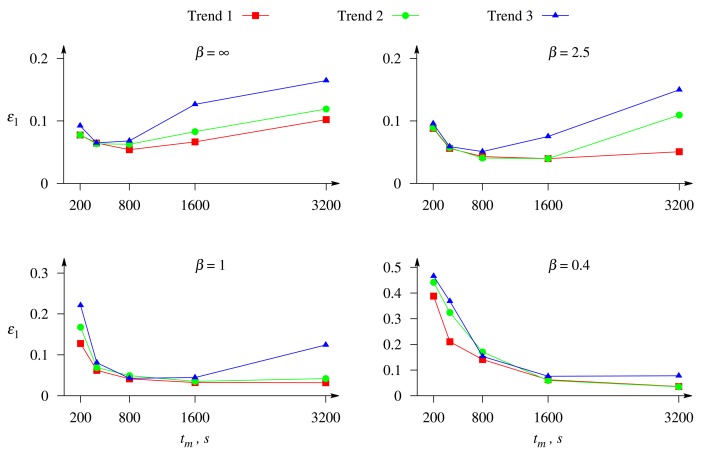
The relative error, *ε*_1_, of the evaluation of the concentration, *c*_1_, *versus* the duration, *t_m_*, of the biosensor operation at different values of the Biot number, *β*, when the response is affected by white Gaussian noise with *σ* = 0.05 and the exponential trend of a different rate. The other parameters are as in Figure 2.

Figure 6 presents the relative error, *ε*_1_, of the estimate, *c̃*_1_, of the substrate, S_1_, *versus* the duration, *t_m_*, of the biosensor action. The error, *ε*_1_, was calculated for different values of the Biot number, *β*, when the biosensor response is affected by white Gaussian noise with *σ* = 0.05 (see Equation (14)) in conjugation with the exponential trend (see Equations (13) and (18)). The biosensor current was calculated as defined in Equation (15). In Figure 6, different curves correspond to the different rates of the exponential trend (see Table 1), and different plots to the different Biot number, *β*.

One can see in Figure 6 that the duration of the biosensor transient response noticeably influences the precision of the concentration evaluation. The influence depends on the rate of the trend: a higher rate leads to a higher impairment of the evaluation precision. On the other hand, the usage of a relatively short biosensor response also leads to a significant impairment of the evaluation precision. Therefore, the duration of the biosensor action can be optimized when the biosensor response is affected by the exponential trend. As one can see from Figure 6, the optimal duration of the response depends on the relative thickness (Biot number *β*) of the outer diffusion layer: a thicker layer (smaller *β*) requires a longer biosensor response; it is worth using *t_m_* = 400 when *β* = ∞, *t_m_* = 800 when *β* = 2.5, *t_m_* = 1,600 when *β* = 1 and *t_m_* = 3, 200 s (steady-state response), when *β* = 0.4.

**Figure 7.**
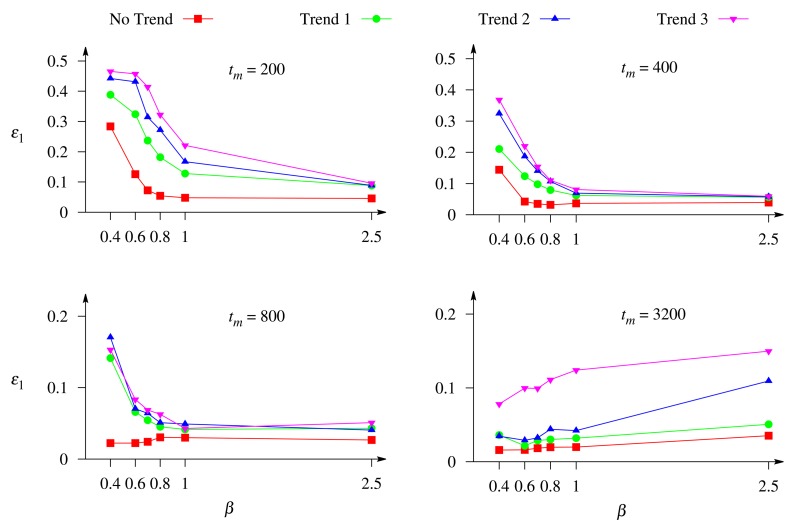
The relative error, *ε*_1_, of the evaluation of the concentration, *c*_1_, of the substrate, S_1_, *versus* the Biot number, *β*, and the duration, *t_m_*, of the biosensor operation when the response is affected by white Gaussian noise with *σ* = 0.05 and the exponential trend of a different rate. The other parameters are as in Figure 2.

Figure 7 shows the dependence of the precision of the concentration evaluation on the Biot number, *β*. Different curves corresponds to a different rate of exponential trend, and different images to a different duration of the biosensor response, *t_m_* ∈ {200, 400, 800, 3, 200} s. The duration of 3,200 s corresponds to the steady-state response in all the numerical simulations of the biosensor response.

One can see in Figure 7 that the relative error, *ε*_1_, of the evaluation of the substrate concentration as a function of the Biot number can be of a different monotonicity. The error, *ε*_1_, is a monotonous decreasing function of the Biot number, *β*, in the case of short-term responses (*t_m_* ∈ {200, 400} s), while in the case of long-term responses (*t_m_* ∈ {800, 3, 200} s), the monotonicity of the function, *ε*_1_(*β*), depends on the trend rate.

Only the dynamics of the biosensor current up to the steady state contains the full information on the dynamics of the biosensors operation. A shorter evolution of the biosensor current contains limited information used in the evaluation of the substrate concentration. The duration of the biosensor operation is especially important at the external diffusion limitation, *i.e.*, a relatively thick outer diffusion layer (small values of the Biot number). On the other hand, a longer response is more distorted by the exponential trend than a shorter one (see Figure 2). Figure 7 shows that the greatest values of the error, *ε*_1_, of the concentration evaluation appear in the case of a thick diffusion layer (small values of *β*) when short-term responses are used in the evaluation.

Increasing the thickness of the external diffusion layer creates an additional diffusion limitation to the substrates, *i.e.*, leads to a lowering of the substrate concentration in the enzyme layer and, thereby, increasing the biosensor sensitivity, as well as prolonging the calibration curve of the biosensor [23,24]. This feature can be also noticed in Figure 7. When the response is not affected by the trend and the long-term response is analyzed (*t_m_* ∈ {800, 3, 200} s), the error, *ε*_1_, of the concentration evaluation decreases with decreasing the Biot number, *β* (increasing the thickness *δ* of the diffusion layer).

Figure 7 also clearly shows that at different conditions, the concentrations are more accurately evaluated when the response is not affected by a trend rather than affected by a trend.

### 4.3. Impact of Maximal Enzymatic Rates

In the numerical experiments discussed above, the values *V*_1_ = 5 × 10^−7^ and *V*_2_ = 5 × 10^−6^ M/s of the maximal enzymatic rates were chosen so that the biosensor response would be under mixed control, controlled by the enzyme kinetics for the first component ( $\Phi_{1}^{2}\approx 0.67<1$) and by the mass transport for the second one ( $\Phi_{2}^{2}\approx 6.7>1$). In order to investigate the impact of the maximal enzymatic rates to the precision of the evaluation, the following two additional configurations of the parameters have been investigated:

- the biosensor response to both substrates is controlled by the enzyme kinetics (*V*_1_ = 5 × 10^−8^, *V*_2_ = 5 × 10^−7^M/s, $\Phi_{1}^{2}\approx 0.067$, $\Phi_{2}^{2}\approx 0.67$);
- the biosensor response to both substrates is controlled by the mass transport (*V*_1_ = 5 × 10^−6^, *V*_2_ = 5 × 10^−5^ M/s, $\Phi_{1}^{2}\approx 6.7$, $\Phi_{2}^{2}\approx 67$).

The calculations showed that the difference between the evaluation precisions of different substrates changes with the changing of the maximal enzymatic rates, while the concentration, *c*_2_, of the second substrate, S_2_, was evaluated notably more precisely in all the numerical experiments discussed above.

**Figure 8.**
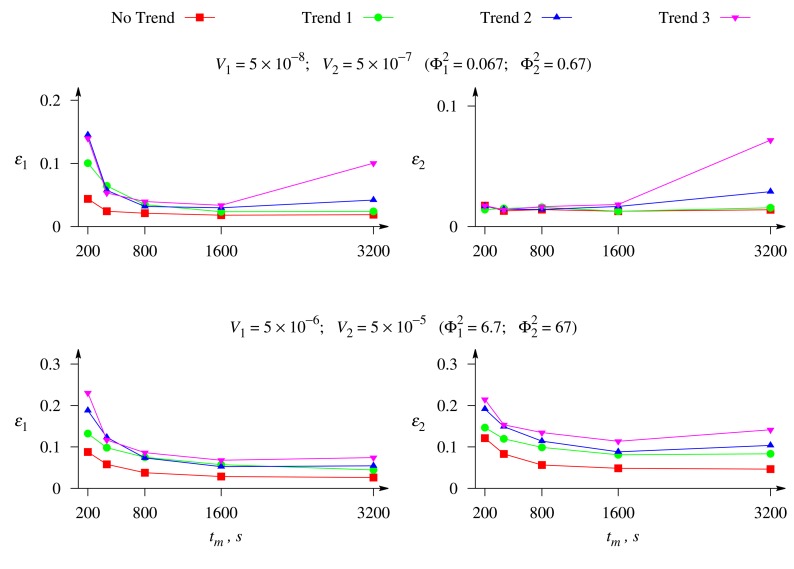
The relative errors, *ε*_1_ and *ε*_2_, of the evaluation of the concentrations, *c*_1_ and *c_2_*, of both substrates, S_1_ and S_2_, *versus* the duration, *t_m_*, of the biosensor operation at different values of the maximal enzymatic rates, *V*_1_ and *V*_2_, and the Biot number *β* = 1, when the response is affected by white Gaussian noise with *σ* = 0.05 and the exponential trend of a different rate. The other parameters are as in Figure 2.

Figure 8 shows the relative errors, *ε*_1_ and *ε*_2_, of the evaluation of the concentrations of both substrates, S_1_ and S_2_, *versus* the duration, *t_m_*, of the biosensor operation at different values of the maximal enzymatic rates, *V*_1_ and *V*_2_, and a moderate value of the Biot number *β* = 1. The response was affected by white Gaussian noise with *σ* = 0.05 and by the exponential trend of a different rate.

As one can see in Figure 8, when the biosensor response to both substrates is controlled by the enzyme kinetics (upper plots), the relative error, *ε*_1_, of the concentration evaluation for the substrate, S_1_, is noticeably greater than the error, *ε*_2_, for the second substrate, S_2_. This is especially noticeable for short durations of the biosensor operation, *t_m_* < 800 s. A similar property of the concentration evaluation was noticed also in the case when the response was under mixed control (see Figure 5). However, when the biosensor response to both substrates is controlled by the mass transport (lower plots), the evaluation errors are rather similar for the both substrates, especially at short durations of the biosensor operation, and even the error, *ε*_2_, becomes slightly greater than the error, *ε*_1_, when long-term responses are used in the concentration evaluation.

Figure 8 also shows a noticeably greater error of the substrate evaluation when the biosensor response to the both substrates is controlled by the mass transport (lower plots).

It is known that when the response of a catalytic biosensor is considerably controlled by the mass transport, the steady-state current practically does not depend on the maximal enzymatic rate (total concentration of enzyme) [26,43]. This feature of amperometric, as well as potentiometric biosensors is notably affected by the relative thickness (Biot number) of the outer diffusion layer. Therefore, when the biosensor response to both substrates of the mixture is controlled by the mass diffusion ( $\Phi_{1}^{2}>1$, $\Phi_{2}^{2}>1$), the impacts of the both substrates on the response become approximately the same, which surely impairs the precision of the concentration evaluations. Choosing the individual maximal enzymatic rates of compounds most appropriate for high precision evaluation of the concentrations is the subject of our future investigation.

The maximal enzymatic rate, *V*_1_, is actually a product of two parameters: the catalytic constant, *k*_3_, introduced in Equation (1a), and the total concentration, *E*_0_, of the enzyme, and correspondingly, *V*_2_ is a product of *E*_0_ and the constant, *k*_4_, introduced in Equation (1b), *V*_1_ = *k*_3_*E*_0_, *V_2_* = *k*_4_*E*_0_ [1,31]. Since, in actual applications, it is usually impossible to change the values of the constants, *k*_3_ and *k*_4_, the maximal rates, *V*_1_ and *V*_2_, as well as the diffusion modules, $\Phi_{1}^{2}$ and $\Phi_{2}^{2}$, might be changed by changing the total concentration, *E*_0_, of the enzyme in the enzyme layer.

## 5. Conclusions

The optimization-based method of the quantitative analysis of the biosensor response proposed in [22] is suitable for the evaluation of the concentrations of the competitive substrates from the biased response of mono-enzyme amperometric biosensors utilizing the Michaelis-Menten kinetics Equation (1) at different diffusion limitations.

The mathematical model, Equations (3)–(9), can be used to simulate pseudo-experimental biosensor responses to mixtures of substrates for evaluating the precision of the quantitative analysis, as well as for calibrating an analytical system.

If the biosensor signal is not affected by the temperature-induced trend, then the substrate concentrations are most accurately evaluated from the biosensor transient response recorded up to a steady state. Shortening the duration of the biosensor operation reduces the accuracy of the evaluation, especially in the case of a relatively thick outer diffusion layer (small values of the Biot number, β; Figure 5). However, if the biosensor response is affected by the induced exponential trend, then the preferable duration of the biosensor action depends on the relative thickness of the outer diffusion layer (Figures 6, 7–8).

At different diffusion limitations and durations of the biosensor operation, the concentrations are more accurately evaluated when the response is not affected by a trend rather than affected by an induced exponential trend (Figures 7 and 8).

At different durations of the biosensor operation and rates of the induced trend, the concentrations of the substrates are more accurately evaluated when the biosensor response to the substrates is controlled by the enzyme kinetics rather than the response being controlled by the mass transport (Figure 8).

## Figures and Tables

**Table 1.** The values of the parameters of the exponential trend Equation (13) substantiated in [22].

**Title**	*E_a_* **(cal/mol)**	*a* **(K/s)**
Trend 1	6,000	3.33 × 10^−3^
Trend 2	24,000	3.33 × 10^−3^
Trend 3	24,000	6.66 × 10^−3^
